# Thirst and drinking in North American watersnakes (*Nerodia* spp.)

**DOI:** 10.1242/jeb.241414

**Published:** 2021-03-05

**Authors:** Matthew Edwards, Coleman M. Sheehy, Matthew T. Fedler, Harvey B. Lillywhite

**Affiliations:** 1 Department of Biology, University of Florida, Gainesville, FL 32611-8525, USA; 2 Florida Museum of Natural History, University of Florida, Gainesville, FL 32611-8525, USA; 3 Fish and Wildlife Research Institute, Florida Fish and Wildlife Conservation Commission, 1105 SW Williston Road, Gainesville, FL 32601, USA

**Keywords:** Dehydration, Snakes, Thirst sensitivity, Water, Water balance, Osmoregulation

## Abstract

We quantified drinking behavior in three species of North American watersnakes: *Nerodia clarkii*, which is a marine or brackish water amphibious species, and *Nerodia* *fasciata* and *Nerodia* *taxispilota*, both freshwater amphibious species. All three species have relatively small and similar thresholds of dehydration (TH, approximately −4% loss of body mass) that elicit thirst and drinking of fresh water. These species have higher thirst sensitivity than several species of hydrophiine and laticaudine sea snakes, which are characterized by much lower TH (greater dehydration, −9% to <−20%). *Nerodia clarkii*, which is often found in coastal oceanic water, refused to drink seawater, but drank fresh water when dehydrated. In separate trials involving dehydration of *N. clarkii* and *N. fasciata* that were concurrently fed fish at regular intervals, snakes eventually refused to eat at TH of approximately −12% of original body mass, but resumed eating after they were allowed to drink fresh water and rehydrate. The drinking behaviors of *Nerodia* corroborate previous data on the importance of fresh water for drinking, and they complement growing evidence that dietary water does not itself mitigate dehydration in snakes. These new data increase understanding of water relationships in the context of evolutionary transitions from land to sea, and they emphasize the importance of fresh water resources in the conservation of coastal and marine species of reptiles.

## Abstract (precis)

**Summary:** Relatively small levels of dehydration elicit drinking of fresh water in three species of North American watersnakes, including a semi-marine species in which moderate, progressive dehydration is also shown to inhibit feeding.

## INTRODUCTION

Like other vertebrates, snakes – including marine species – utilize, and may require, various sources of free water in their environment to remain in water balance (Murphy and DeNardo, 2019; Rash and Lillywhite, 2019; Sandfoss and Lillywhite, 2019). Because of their elongated body form and occurrence in a broad range of habitats, snakes are especially well suited for investigations of how and when water is procured to replace losses and to supplement dietary and metabolic water that is acquired independently of drinking. Although snakes are generally well adapted to conserving water and may drink infrequently, drinking fresh water (FW) likely remains necessary for water homeostasis in most species including those inhabiting desiccating environments (Bonnet and Brischoux, 2008; Lillywhite et al., 2008, 2014a,b, 2015, 2019; Lillywhite, 2017; Murphy and DeNardo, 2019).

Previous studies of dehydration thresholds (TH) that elicit thirst and drinking of FW have been conducted largely with marine snakes, which show variable responses but do not drink seawater (SW) and require FW for water balance (Lillywhite et al., 2008, 2012, 2014a,b, 2015, 2019). Indeed, some species of sea snakes drink intensely when FW becomes available following drought (Bonnet and Brischoux, 2008; Lillywhite et al., 2019), as also occurs in some species of lizards inhabiting deserts (Davis and DeNardo, 2007). While the prey of many reptiles generally contains 60–75% water, dietary water does not appear to satisfy water requirements when sources of free drinking water are scarce or absent (Lillywhite et al., 2008, 2014b; Murphy and DeNardo, 2019). Moreover, snakes increase drinking following meal consumption, which suggests that feeding increases (rather than diminishes) the requirement for body water (Lillywhite, 2017).

Here, we report new data for TH determined from trials of graded dehydration in three species of North American watersnakes: *Nerodia clarkii* (Baird and Girard 1853), a semi-marine, amphibious and estuarine species; and *Nerodia* *fasciata* (Linnaeus 1766) and *Nerodia* *taxispilota* (Holbrook 1838), both characteristic and common semi-aquatic species of freshwater habitats. In some *N. clarkii* and *N. fasciata*, we also determined the level of dehydration at which snakes stopped feeding, a topic not previously investigated with quantitative data.

## MATERIALS AND METHODS

All snakes were acquired opportunistically by hand-capture of wild specimens. *Nerodia clarkii* (*n*=19; mean±s.e.m. mass 48.5±19.3 g) were captured from saltmarsh habitats at Seahorse Key, Levy County, FL, USA. Snakes of the other species (*n*=22 *N. fasciata*, mass 213.7±32.2 g; *n*=5 *N. taxispilota*, mass 222.5±52.7 g) were hand-captured in creeks and swampy habitats in Alachua County, FL, USA. Snakes were brought to the laboratory at the University of Florida, where they were maintained in Plexiglas cages with newspaper substrate and a water bowl that was withheld only during periods of experimental dehydration (see below). Snakes were allowed to drink, urinate and defecate over the course of several days to reach a stable body mass, which was used as the respective baseline measurement.

To determine the TH at which snakes first drank FW, individual snakes were dehydrated in air following progressive protocols we have used previously (Lillywhite et al., 2008, 2012, 2014a,b). Each animal was held for short periods without access to water inside fiberglass cages with a mesh window at the top to allow air circulation and a newspaper cover over the floor. Each animal was weighed periodically to determine the rate and quantity of mass lost. Mass measurements were repeated at intervals of several hours or 10–12 h overnight. Following intervals of dehydration (roughly equal to <2% body mass), each animal was tested for drinking. Each snake was placed separately in a tilted cage (without newspaper) with a pool of tap water at one end for drinking during a 1 h period. In most cases, the head of the snake was gently lowered to the water, and it drank immediately. Before weighing, each snake was carefully dried on a towel to remove excess water on the skin, then weighed before and after each period with access to water. Snakes were weighed in a tared container to within 0.1 g using a triple beam or electronic balance.

Four *N. clarkii* were dehydrated to between 2% and ≤10% of initial mass and offered SW to drink. None of the snakes drank SW, so these were then offered FW. Because freshwater species of watersnakes naive to SW have been shown to consume it and die as a result (Dunson, 1980), we did not offer SW to *N. fasciata* or *N. taxispilota*.

In separate tests, five *N. clarkii* and five *N. fasciata* were kept without access to water and continuously dehydrated while offered pre-weighed, live feeder fish (*Danio rerio*) every 3 days. During feedings, multiple live fish were dropped onto a dry substrate (inside the cage near the snake), where they flipped themselves and moved erratically. Such movements were previously shown to be a strong stimulus attracting snakes to the prey. If a snake refused to eat, it was offered food again (usually 3 times) before being allowed to rehydrate by drinking FW. Snakes were again offered food following rehydration to the baseline mass. The mass of each snake was measured daily with some exceptions for short periods when the investigators were absent.

All snakes were released at, or very near, their site of capture at various times over the entire course of the experimental measurements. All snakes appeared normally hydrated and in healthy condition before their release.

### Statistics and analysis

All measurements are expressed as means±s.e.m. Comparisons of species means were made using ANOVA, and *t*-tests were used to compare means of apparent dehydration thresholds (ATH, see below) in the two species used in the dehydration and feeding trials. Statistical analyses were conducted using Statview, SAS, version 5.0.1.0, and the level of significance was considered to be *P*≤0.05.

### Ethics and welfare

This research was conducted within guidelines and approval of the University of Florida IACUC, #201802798, and with approval of the US Fish and Wildlife Service (permit #41511-12-10).

## RESULTS AND DISCUSSION

**Fig. 1.**
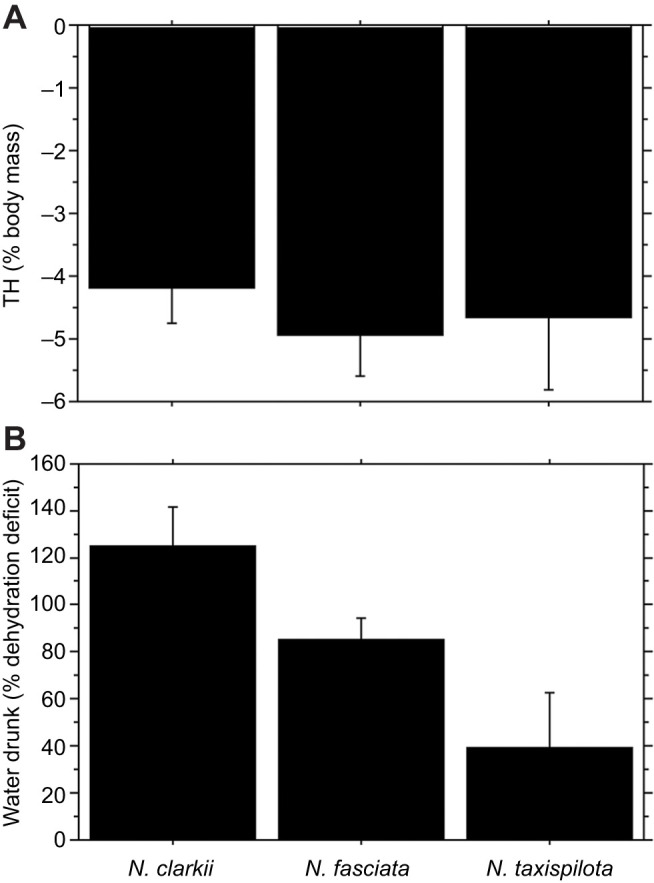
**Dehydration threshold (TH) in three species of North American watersnakes of the genus *Nerodia*.** (A) Mean dehydration threshold for thirst or freshwater drinking, expressed as a negative percentage of initial body mass that is lost during dehydration. The bars are statistically similar. (B) Mean amount of water that snakes drank at TH, as a percentage of the dehydration deficit. The bars are significantly different. Lines associated with bars represent 1 s.e.m., and further details concerning statistics can be found in Results and Discussion.

All snakes drank FW when induced by relatively small amounts of dehydration. No *N. clarkii* that was tested drank SW. The mean TH that induced drinking of FW was −4.2±0.6% body mass in *N. clarkii* (*n*=19), −4.9±0.6% body mass in *N. fasciata* (*n*=22) and −4.7±1.2% body mass in *N. taxispilota* (*n*=5). The mean TH was statistically similar among the three species (ANOVA, *F*_2,43_=0.373, *P*=0.6909) (Fig. 1A). The mean TH for individual snakes (intra-individual variation) ranged from −1.2 to −11.4% original body mass in *N. clarkii*, from −1.4 to −10.7% in *N. fasciata*, and from −1.9 to −7.7% in *N. taxispilota*. All snakes drank variable amounts of water, sometimes over- or under-drinking with respect to replenishing the deficit of dehydration. The mean amount of water that was drunk at TH, expressed as a percentage of the dehydration deficit, was 124.8±16.9% (*n*=19) in *N. clarkii*, 84.9±9.1% (*n*=22) in *N. fasciata* and 39.0±23.2% (*n*=5) in *N. taxispilota*. These means were statistically different (ANOVA, *F*_2,43_=5.112, *P*=0.0102) (Fig. 1B). Snakes were observed during offerings of FW and, characteristically, they drank immediately when placed where the tongue contacted water during tongue flicking. A summary of dehydration TH and drinking data is shown in Table 1 and Fig. 1.

**Table 1.**
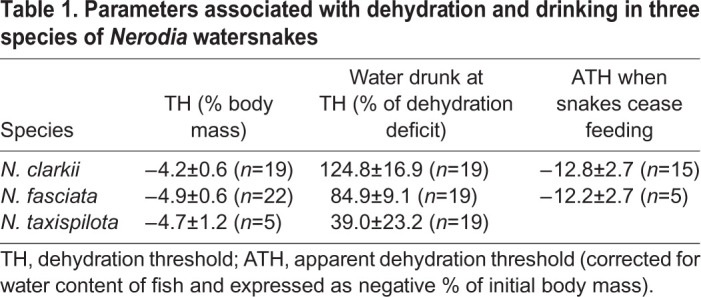
**Parameters associated with dehydration and drinking in three species of *Nerodia* watersnakes**

Five each of *N. clarkii* and *N. fasciata* were kept without water but allowed to consume fish. These snakes readily ate while hydrated, but eventually stopped feeding during prolonged dehydration with repeated access to food. To express the dehydration that was tolerated before snakes stopped feeding, the mass of fish that were eaten during the trials must be taken into account. Therefore, we calculated the ATH as *M*_o_−(*M*_f_−*M*_fish_), where *M*_o_ is the hydrated mass of the snake at the beginning of the dehydration, *M*_f_ is the dehydrated mass of the snake at the point when it refused to eat fish, and *M*_fish_ is the mass of fish that were eaten by the snake during the dehydration trial. Calculated ATH at which snakes lost interest in feeding is, of course, an over-estimate of dehydration because of water that is gained from fish that were eaten during the dehydration trials preceding the point at which they refused to eat. To crudely account for this, the water composition of fish was assumed to equal 71% (Thorson, 1961), and this percentage of the mass of fish was assumed to contribute to the water balance of dehydrating snakes and therefore was not subtracted from the mass of snakes. Hence, in this case (correction for body water of ingested fish) the corrected ATH=*M*_o_−(*M*_f_−0.29*M*_fish_).

**Fig. 2.**
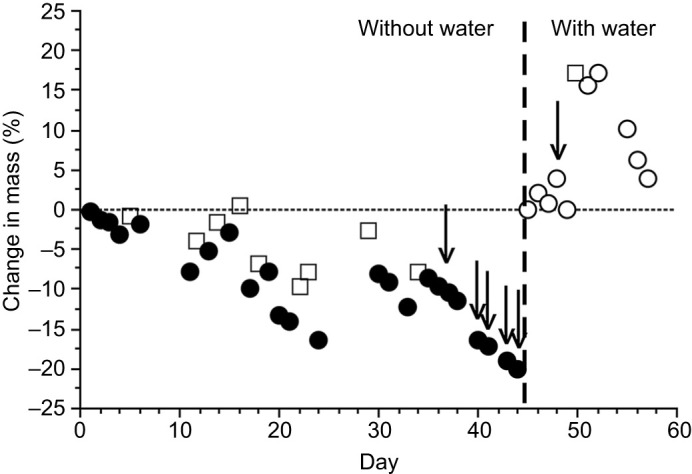
**Plot of relative changes of body mass in a 15.6 g watersnake (*Nerodia clarkii*).** This snake was dehydrated with intermittent feeding but without access to water (left of vertical dashed line). Arrows indicate when the snake refused food; it resumed feeding following rehydration by drinking. Filled circles indicate the mass of the snake while dehydrating; open circles indicate the mass of the snake while it had access to fresh water (right of vertical dashed line); open squares indicate the mass of the snake following voluntary ingestion of live fish. Note that at the end of the dehydration period (vertical dashed line), the snake did not eat immediately (day 48), but began feeding (day 50) after a few days when drinking had restored its starting mass. The mass of the snake was not measured on days without data points.

The mean ‘corrected’ ATH was −12.8±2.7% (*n*=5) in *N. clarkii* and −12.2±2.7% (*n*=5) in *N. fasciata* (Table 1). The ATH values in the two species were not statistically different (*t*-test, *P*=0.8766, d.f.=8). An example plot of feeding and drinking behavior in these experiments is illustrated in Fig. 2.

### Conclusions and perspectives

Sources of water that contribute to homeostasis of body water in vertebrates include dietary water, metabolic water and free drinking water in the environment. Investigations of water balance have related largely to ionic and osmotic regulation of body fluids and excretory function, with less attention given to drinking behaviors and environmental sources of water (Rash and Lillywhite, 2019). Animals living in deserts and marine environments are of particular interest because of the scarcity of free water and selective forces that favor the conservation of dietary and metabolic water. Patterns of precipitation and drought are also a present and future concern related to climate change (Trenberth, 2011; Dai et al., 2018).

Recent studies have demonstrated that various species of marine snakes do not drink SW, as formerly thought, but instead rely on FW (e.g. from oceanic freshwater lenses formed by rainfall) to remain in water balance, and dehydrate at sea when there is no rainfall (Bonnet and Brischoux, 2008; Lillywhite et al., 2008, 2012, 2014a,b, 2015, 2019). Among the suite of adaptations by which these snakes survive in marine environments is a high tolerance to dehydration and hence a relatively large TH (Lillywhite et al., 2012, 2015). Considering the utilization of FW by marine snakes, coupled with a relatively large TH for thirst and drinking, the sensitivity to dehydration and drinking behaviors of other taxa having semi-marine or semi-aquatic habits are of comparative and evolutionary interest. Here, we demonstrate that the TH for FW drinking in three species of *Nerodia* is around −4% loss of body mass, whereas similar mean TH for marine snakes ranges from around −7% in marine file snakes, *Acrochordus granulatus* (Lillywhite et al., 2014a), and −9% in amphibious, oviparous sea kraits (Lillywhite et al., 2008), to <−20% in species of fully marine, viviparous hydrophiine sea snakes (Lillywhite et al., 2015). Thus, freshwater and semi-marine *Nerodia* (both viviparous) are more sensitive to dehydration as it stimulates thirst and drinking (Table 1, Fig. 1).

The similarity of TH among the three species of *Nerodia* was surprising insofar as *N. clarkii* is far more marine in habit than are the other two species, *N. fasciata* and *N. taxispilota*. Dunson (1980) reported that semi-marine *N. clarkii* has a lower skin permeability to both water and sodium than does *N. fasciata*, which occurs in freshwater habitats. Hence, Dunson (1980) concluded that the distinction between freshwater and estuarine populations of *Nerodia* is not simply behavioral but also involves physiological differences. It appears in light of the present data, however, that *N. clarkii* has not at this time evolved any differences in thirst and drinking threshold that are distinct from those of *N. fasciata* and *N. taxispilota*. Phylogenetically, *N. clarkii* and *N. fasciata* are sister taxa, while *N. taxispilota* is one of the more distantly related members of the genus (McVay et al., 2015).

Natricine snakes include coastal and semi-marine species (Dunson, 1980; Dunson and Mazzotti, 1989; Brischoux et al., 2017) but represent a phylogenetic lineage that is distinct from that of sea snakes (Elapidae). *Nerodia clarkii* is semi-marine and spends much of its time in salt marsh or shallow ocean water. However, unlike the completely marine sea snakes, *N. clarkii* has access to terrestrial sources of FW including precipitation that forms temporary pools and drips from vegetation (see Bonnet and Brischoux, 2008). We suggest that a reduction in the TH of the freshwater drinking response in *N. clarkii* likely has not occurred for this reason. In the population from which we captured individuals for this study, snakes spend time in salt marsh surrounding numerous small islands comprising the Cedar Keys, but they also come ashore and are commonly found in grasses, mangroves and insular hammock habitats. Other taxa of terrestrial snakes representing three families (Colubridae, Elapidae, Viperidae) also drink FW following just a few percent loss of body mass, similarly to *Nerodia* spp. (H.B.L., unpublished data; Sandfoss and Lillywhite, 2019). Moreover, the amphibious sea kraits (Elapidae), which also have access to terrestrial sources of FW, have greater thirst sensitivity and a TH (−7% body mass) that is closer to the values reported here than to the values for hydrophiine (fully marine) sea snakes (Lillywhite et al., 2008, cf. Lillywhite et al., 2015). Thus, we suggest that suppression of thirst and high tolerance of dehydration before drinking appear to reflect evolutionary adjustment to totally marine habits and not simply contact with SW.

Theoretical (Lillywhite et al., 2008) and preliminary data (Lillywhite et al., 2014a, 2019) suggest that prolonged dehydration inhibits feeding. Moreover, snakes representing four species – including *N. fasciata* – increase consumption of water following feeding (Lillywhite, 2017), further suggesting that eating creates a net loss, rather than gain, of water. Thus, we tested a subsample of snakes and here demonstrate that moderate dehydration (12% loss of body mass) with access to, and consumption of, prey inhibits feeding (Fig. 2). Such a response makes sense only if consumption of food renders a net loss, rather than gain, of water (see also Lillywhite et al., 2008, 2014a; Wright et al., 2013; Lillywhite, 2017; Murphy and DeNardo, 2019). Gains of dietary water in food are evidently offset by losses of water attributable to digestion and excretion of associated waste products (Lillywhite et al., 2008). Hence, consumption of food can actually exacerbate the dehydration associated with drought, instead of providing a net positive benefit. Importantly, this information suggests an additional mechanism by which drought might impact energetics (consumption of food) as well as water homeostasis per se. Consistent with these results, xerophylic rattlesnakes (*Crotalus lepidus*) experience negative water balance (efflux>influx) when consuming prey (Beaupre, 1996). In desert-inhabiting *Crotalus atrox*, increases in plasma osmolality were similar between moderately dehydrated snakes that did or did not consume a meal; moreover, snakes that received a meal while dehydrating reached a state of severe dehydration more than a week earlier than did snakes without food (Murphy and DeNardo, 2019).

In summary, semi-aquatic freshwater and marine species of *Nerodia* are characterized by relatively small dehydration thresholds that elicit FW drinking, in comparison with known thresholds for marine species of sea snakes. The new data reported here are significant for understanding water relationships in the context of evolutionary transitions from land to sea. Although secondarily marine snakes remain dependent on sources of FW for drinking, suppression of sensitivity to thirst evidently requires long-term exposure to strictly marine environments (see also Brischoux et al., 2017). Our studies also indicate that the location and persistence of FW sources are important considerations in planning for conservation of coastal species of reptiles. We emphasize the importance of sources of FW for drinking because dietary water does not itself mitigate dehydration.
